# Standardizing the experimental conditions for using urine in NMR-based metabolomic studies with a particular focus on diagnostic studies: a review

**DOI:** 10.1007/s11306-014-0746-7

**Published:** 2014-11-21

**Authors:** Abdul-Hamid Emwas, Claudio Luchinat, Paola Turano, Leonardo Tenori, Raja Roy, Reza M. Salek, Danielle Ryan, Jasmeen S. Merzaban, Rima Kaddurah-Daouk, Ana Carolina Zeri, G. A. Nagana Gowda, Daniel Raftery, Yulan Wang, Lorraine Brennan, David S. Wishart

**Affiliations:** 1 Imaging and Characterization Core Lab, King Abdullah University of Science and Technology, KSA, Thuwal, Saudi Arabia; 2 Biological and Environmental Sciences and Engineering, King Abdullah University of Science and Technology, KSA, Thuwal, Saudi Arabia; 3 Centro Risonanze Magnetiche – CERM, University of Florence, Florence, Italy; 4 FiorGen Foundation, 50019 Sesto Fiorentino, Florence, Italy; 5 Centre of Biomedical Research, Formerly known as Centre of Biomedical Magnetic Resonance, Sanjay Gandhi Post-Graduate Institute of Medical Sciences Campus, Lucknow, India; 6 Department of Biochemistry & Cambridge Systems Biology Centre, University of Cambridge, Cambridge, UK; 7 European Molecular Biology Laboratory, European Bioinformatics Institute (EMBL-EBI), Wellcome Trust Genome Campus, Cambridge, CB10 1SD UK; 8 School of Agricultural and Wine Sciences, Charles Sturt University, Wagga Wagga, Australia; 9 Pharmacometabolomics Center, School of Medicine, Duke University, Durham, USA; 10 Brazilian Biosciences National Laboratory, LNBio, Campinas, SP Brazil; 11 Department of Anethesiology and Pain Medicine, Northwest Metabolomics Research Center, University of Washington, 850 Republican St., Seattle, WA 98109 USA; 12 Wuhan Institute of Physics and Mathematics, Chinese Academy of Sciences, Beijing, China; 13 Institute of Food and Health and Conway Institute, School of Agriculture & Food Science, Dublin 4, Ireland; 14 Department of Computing Science, University of Alberta, Edmonton, Alberta Canada

**Keywords:** NMR, Metabolomics, Metabonomics, Metabolites profiling, Urine, Biomarker, Human diseases, Standardization, Diagnosis, Recommendations

## Abstract

The metabolic composition of human biofluids can provide important diagnostic and prognostic information. Among the biofluids most commonly analyzed in metabolomic studies, urine appears to be particularly useful. It is abundant, readily available, easily stored and can be collected by simple, noninvasive techniques. Moreover, given its chemical complexity, urine is particularly rich in potential disease biomarkers. This makes it an ideal biofluid for detecting or monitoring disease processes. Among the metabolomic tools available for urine analysis, NMR spectroscopy has proven to be particularly well-suited, because the technique is highly reproducible and requires minimal sample handling. As it permits the identification and quantification of a wide range of compounds, independent of their chemical properties, NMR spectroscopy has been frequently used to detect or discover disease fingerprints and biomarkers in urine. Although protocols for NMR data acquisition and processing have been standardized, no consensus on protocols for urine sample selection, collection, storage and preparation in NMR-based metabolomic studies have been developed. This lack of consensus may be leading to spurious biomarkers being reported and may account for a general lack of reproducibility between laboratories. Here, we review a large number of published studies on NMR-based urine metabolic profiling with the aim of identifying key variables that may affect the results of metabolomics studies. From this survey, we identify a number of issues that require either standardization or careful accounting in experimental design and provide some recommendations for urine collection, sample preparation and data acquisition.

## Introduction

Human diseases lead to pathophysiological changes that, in turn, lead to variations in the concentrations of metabolites in tissues and biological fluids. Metabolomics is a powerful approach that permits the global monitoring of metabolite levels when a living system responds to environmental effects, physiological stimuli and/or genetic modifications. As a result, metabolomics represents a powerful technique for disease fingerprinting and biomarker discovery and for the identification of perturbed biochemical pathways. Furthermore, metabolomic data can be integrated with genomic, transcriptomic and proteomic data to improve our understanding of disease etiology and thereby optimize treatment interventions (Yizhak et al. 2010; Griffin 2006; Tang et al. 2009; Nambiar et al. 2010; Rantalainen et al. 2006). In many cases, the changes in urine metabolite concentrations induced by human diseases can be greater than the changes seen in protein levels, giving metabolomics an extra diagnostic advantage (Kell 2006; Urbanczyk-Wochniak et al. 2003; Raamsdonk et al. 2001; Cascante et al. 2002). Thus, measuring metabolite profiles in human urine has the potential to be used as an effective method to diagnose a number of diseases and to monitor an individual’s general health status. Indeed, urine represents an ideal biofluid for metabolomics studies because it captures and concentrates a wide range of metabolites and metabolic processes occurring throughout the body. As a consequence, urine is particularly useful in providing specific biomarkers for disease, toxicity and drug metabolism. Urine’s non-viscous character combined with the fact that it can be obtained non-invasively, in large quantities and that it contains fewer protein complexes and lipids than other body fluids (such as blood) has long made it a favored biofluid for many metabolomics applications.

Metabolite profiles in biofluids may be measured using several techniques including high performance liquid chromatography (Al-Talla et al. 2011a, b; Li et al. 2013; Nie et al. 2014; Szultka et al. 2014; Lan et al. 2010; Liang et al. 2010; Zheng et al. 2010), tandem mass spectrometry (Guo et al. 2013; Huang et al. 2013b; Vadla et al. 2013; Allard et al. 2008; Chen et al. 2009; Cho et al. 2009), gas chromatography with mass spectrometry (Karamani et al. 2013; Zheng et al. 2013; Jobu et al. 2012; Weusten et al. 2012; Xie et al. 2012; Kumar et al. 2010; Qiu et al. 2010; Sun et al. 2009) and NMR spectroscopy (Kim et al. 2013; Wang et al. 2013a; Bertram et al. 2011; Duarte and Gil 2012; He et al. 2012; Calvani et al. 2010; Liu et al. 2010; Zhang et al. 2010; Michell et al. 2008; Wishart 2008; Zhang et al. 2008). Each approach has its own advantages and disadvantages, with some techniques being better for analyzing certain biofluids than others. NMR, in particular, permits the visualization of thousands of distinct peaks and enables the detection and quantification of more than 200 compounds in human urine (Bouatra et al. 2013). As a consequence, metabolic profiling of human urine by NMR spectroscopy has been used to discover a number of novel biomarkers that can be used for disease diagnosis, prognosis, prediction, and monitoring (Nevedomskaya et al. 2012; Pu et al. 2012; Sachse et al. 2012; Zhang et al. 2012; Carrola et al. 2011; Jung et al. 2011; Sengupta et al. 2011; Maher et al. 2009; Tzovaras et al. 2009).

To date, most metabolomic studies of urine and human disease have been designed as “case–control” studies in which urine samples from patients are compared with healthy controls. However, due to the environmental and genetic diversity of human populations and to day-to-day variability, comprehensive and robust metabolomic analysis of urine is a non-trivial task. In addition, study designs (e.g., single or multiple collections), and especially pre-analytical procedures, are generally not standardized, thereby making inter-laboratory comparisons difficult. Likewise, few studies take advantage of NMR spectroscopy’s ability to obtain a metabolic fingerprint and to accurately determine multiple metabolite concentrations. NMR can also be used to obtain a complete spectral fingerprint of a given biofluid, thereby capturing all of the NMR-detectable signals or features. In this way, it is possible to obtain metabolic information without necessarily knowing the exact composition of the samples or signal assignment, so that multivariate data analyses can be performed on the spectra to build statistical models for a diagnostic/prognostic purpose.

A number of urinary metabolomics studies have reported biomarkers that effectively discriminate between diseased subjects and healthy controls (Dawiskiba et al. 2014; Bernini et al. 2011a; Fanos et al. 2013; Wen et al. 2010; Duarte et al. 2014; Fanos et al. 2012; Weiss and Kim 2012). However, most of the biomarkers described so far have not been validated nor have they been proven to be truly specific to the disease that was studied. Furthermore, the lack of standardized protocols in many urine studies that distinguish patients from healthy controls is potentially leading to erroneous results arising from the significant contributions of major confounding factors such as diet, age and gender. To identify a reliable disease biomarker or to determine a discriminating metabolic fingerprint, it is crucial to control for and understand all factors that may alter the normal composition of human urine, so that normal metabolic fluctuations are not confused with pathological changes. In order to minimize the effect of confounding factors, it is crucial to conduct such studies under standardized conditions that would allow researchers from different groups to compare and validate their results. Most importantly, standardization will allow researchers from different parts of the world to store metabolite data from urine in newly emerging metabolomics databases such as MetaboLights and the Human Metabolome Database (HMDB) (Haug et al. 2013; Salek et al. 2013a, b; Wishart et al. 2007, 2009), which will enable direct comparisons of various studies.

Metabolomic analysis of urine samples has been successfully used to identify a variety of metabolomic fingerprints and potential biomarkers under a range of different conditions. An exhaustive review of this literature is beyond the scope of the present review. Instead, some key examples are highlighted to illustrate the potential power of the approach. An area of extensive research is the study of cancer biomarkers, including the development of putative biomarkers for bladder cancer (Alberice et al. 2013; Zhang et al. 2012), lung cancer (Carrola et al. 2011), prostate cancer (Duarte and Gil 2012; Kumar et al. 2014), liver cancer (Chen et al. 2011), colorectal cancer (Wang et al. 2010), esophageal cancer (Davis et al. 2012), and kidney cancer (Huang et al. 2013c; Weiss and Kim 2012; Kim et al. 2011; Ganti and Weiss 2011). Examples of the metabolites identified as putative biomarkers of kidney cancer include acylcarnitines, quinolinate, gentisate, and 4-hydroxybenzoate. Application of metabolomics in cancer staging has recently emerged and its importance is expected to grow in the coming years (Kim et al. 2011).

Applications of urine metabolomics to the study of inflammatory bowel disease (IBD) has also been successful from a diagnostic viewpoint. A number of studies have reported metabolomic profiles that are capable of separating subjects with IBD subtypes from healthy controls (Schicho et al. 2012). More recently, an NMR based profiling of urine samples demonstrated the ability to distinguish patients with active IBD from those with IBD in remission (Dawiskiba et al. 2014). Other conditions such as heart failure also appear to be detectable through urine-base metabolomics. Proton NMR spectra of the urine of 15 patients with ischemic heart failure (HF) were compared with the proton NMR spectra of the urine of 20 healthy controls. The results show clear separation between the patient samples and healthy ones, where the HF patient samples had higher concentrations of metabolites such as acetate and acetone compared with the healthy control samples. On the other hand, the HF patients’ urine had decreased levels of 1-methylnicotinamide and increased levels of methylmalonic acid, cytosine and phenylacetylglycine compared to the healthy controls (Kang et al. 2011). It was reported that the perturbation of these metabolites could be related to TCA cycle metabolites and fatty acid metabolism (Kang et al. 2011). Applications of the metabolomics-based approach in the field of human toxicology have also yielded positive results. An example is the study of acetaminophen-induced hepatoxicity. A number of studies have identified metabolic signatures associated with hepatoxicity (Kim et al. 2013; Clayton et al. 2006). Overall, the above examples and additional examples included in Table 1 highlight the potential use of metabolomics analysis of urine for disease diagnosis. For progress of this discipline and to avoid the discovery of false positives it is imperative that we have a good understanding of the non-physiological factors influencing metabolite levels in the urine.

**Table 1:** A randomly selected examples of urinary analysis studies using an NMR-based metabolomics approach with particular focus on the variations of some reported experimental conditions such as centrifugation speed, storage temperature and pH values

Centrifuge time	Centrifuge speed/rpm or *g*	Storage temp.	pH	Study description	References
5 min	1,500	−80	7.4	Urine specimens were collected before and at 4 and 24 h after surgery from 106 patients with acute kidney injury and analyzed by means of NMR	(Zacharias et al. 2013)
15	3,000		7.4	Urinary tract infections (UTI) studied by NMR of urine samples using a longitudinal design. The study included four classes of samples originating from controls (N = 35) at day 0 (baseline control), UTI patients (N = 32) at day 0 (baseline), UTI patients (n = 29) at day 4 and UTI patients after recovery from infection (n = 37) at day 30	(Nevedomskaya et al. 2012)
10	13,200	−80	7.4	NMR based metabolomics of urine differentiates dogs with bladder cancer (n = 40) from healthy controls (n = 42)	(J. Zhang et al. 2012)
10 min	3184	−80	7.4	A NMR metabonomics study involving 447 individuals from Uganda, infected by *S. mansoni.* Urines were collected at five time-points, before and after chemotherapy with praziquantel	(Balog et al. 2011)
10 min	12,000	−70	7.0	Patients with systolic HF of ischemic origin (n = 15) and healthy controls (n = 20) where compared using NMR based metabolomics in urine samples	(Kang et al. 2011)
5 min	8,000	−80	7.0	^1^H NMR-based metabonomics was applied to investigate lung cancer metabolic signatures in urine. Urine samples from lung cancer patients (n = 71) and a control healthy group (n = 54) were analyzed by high resolution ^1^H NMR (500 MHz). The classification model showed 93 % sensitivity, 94 % specificity and an overall classification rate of 93.5 %	(Carrola et al. 2011)
10 min	7,000		7.0	In this study, age-related metabolic changes in children of age 12 years and below were analyzed by NMR spectroscopy. Urine samples from 55 children, with no diagnosed disease, were collected and analyzed	(Gu et al. 2009)
10 min	2500	−20	NM	Urine, plasma, and saliva were collected from 30 healthy volunteers (23 females, 7 males) on 4 separate mornings. For visits 1 and 2, free food choice was permitted on the day before biofluid collection. Food choice on the day before visit 3 was intended to mimic that for visit 2, and all foods were standardized on the day before visit 4. Samples were analyzed by using 1H NMR	(M. C. Walsh et al. 2006)
5 min	10,000	−20	7.0	22 women were segregated into an untrained (n = 10) or trained (n = 12) group depending on their physical training background. The subjects performed two exercises in a randomized order: a prolonged exercise test and a short-term, intensive exercise test. Urine specimens were collected before and 30 min after each test. The samples were analyzed by (1)H NMR spectroscopy	(Enea et al. 2010)
5 min	8000	NM	7.4	12 healthy male participants (age range of 25–74 years) consumed three different diets, in a randomized order, for continuous 15-days-periods with an intervening washout period between each diet of 7 days duration. Each participant provided three consecutive 24-h urine collections on days 13, 14, and 15 of each dietary period, and 1H NMR spectra were acquired on all samples	(Stella et al. 2006)
5	13,400	NM	7.4	This study aimed to identify novel markers for gestational diabetes in the biochemical profile of maternal urine using NMR metabolomics. It also studied the general effects of pregnancy and delivery on the urine profile. Urine samples were collected at three time points from 823 healthy, pregnant women and analyzed using (1)H-NMR spectroscopy	(Sachse et al. 2012)
NM	NM	NM	7.4	Changes in metabolism in volunteers living near a point source of environmental pollution were investigated. NMR was used to acquire urinary metabolic profiles from 178 human volunteers	(Ellis et al. 2012)
10	12,000	−80 C	7.4	This study included 16 bilateral ureteral obstruction (BUO) patients and nine unilateral ureteral obstruction (UUO) patients. The obstructions in all of the patients were successfully relieved after treatment. Urine samples at different time points before and after treatment were obtained, and their (1)H NMR spectra were recorded	(Dong et al. 2013)
3 min	13,000	−20	7.0 ± 0.1	NMR-based metabolomics on blood serum and urine samples from 32 patients representative of a total cohort of 1,970 multiple myeloma patients	(Lodi et al. 2013)
NM	NM	Freeze-dried	6.0	Study on the pharmacological effects of rosiglitazone in plasma and urine samples from patients with type 2 diabetes mellitus (n = 16) and healthy volunteers (n = 16)	(van Doorn et al. 2007)
NM	NM	−80	6.8 ± 0.1	Investigations of the effects of gender, diurnal variation, and age in human urinary metabolome. 30 male and 30 female subjects aged 19–69 years, self-identified as healthy, participated in this study. Individuals provided two urine samples a day, one as a first void and a second at ~5 p.m., for four nonconsecutive days	(Slupsky et al. 2007)
5 min	14,000	−80	7.0	About 40 urine samples (first in the morning, preprandial) were collected from 20 healthy individuals (9 males, 11 females) in the age range 25–55 over a period of about 3 months to demonstrated the stability in time of the urinary metabolome	(Bernini et al. 2009)
NM	NM	−80	7.4	84 urine samples were collected from 12 healthy volunteers (7 time points, 8 males and 4 females) and 50 samples from 30 T2DM patients (1–3 time points, 17 males and 13 females)	(Salek et al. 2007)
NM	NM	NM	NM	A NMR based metabolomics investigation of Hepatitis C virus infection identified 32 of the 34 patients in the disease group as positive and 31 of the 32 individuals in the control group as negative, demonstrating 94 % sensitivity and specificity of 97 %	(Godoy et al. 2010)
15 min	6,000	−80	7.4	Urine samples were collected from 119 Sardinian children (63 males, 56 females, average age 8.3 ± 2.9). Of these, 90 were healthy and 29 type 1 diabetic without complications, with average diabetes duration of 5.2 ± 3.9 years	(Culeddu et al. 2012)
NM	NM	−20	4	A total of twenty-four 8-year-old boys were asked to take 53 g protein as milk (n 12) or meat daily (n 12). At baseline and after 7 days, urine and serum samples were collected and high-resolution 1H NMR spectra were acquired on these using a 800 MHz spectrometer	(Bertram et al. 2007a)
5 min	1600	−20	6.0	The plasma and urine metabolome of 192 overweight 12–15-year-old adolescents (BMI of 25.4 ± 2.3 kg/m^2^) were examined in order to elucidate gender, pubertal development, physical activity and intra-/interindividual differences affecting the metabolome detected by proton NMR spectroscopy	(Kochhar et al. 2006)
10	12,000	NM	7.0	Serum and urine samples were collected from 24 patients with ulcerative colitis, 19 patients with the Crohn’s disease and 17 healthy controls	(Dawiskiba et al. 2014)
10	12,000	−80	7.4	Urine samples were collected from 30 children and teenagers aged 4–19 with T1D and 12 healthy children, aged 9, as control group. Patients were divided into two groups according to their level of glycated hemoglobin	(Deja et al. 2013)
10	3,000	−80	7.4	The urinary metabolic profiles of 30 autistic and 28 matched healthy children were obtained using NMR-based approach	(Mavel et al. 2013)
10	20,000	−80	7.4	32 schizophrenia inpatients and 30 healthy volunteers were recruited. Samples of patients were collected at baseline and weeks 3 and 6 during hospitalization. Samples of healthy controls were collected only once following the same procedure	(Cai et al. 2012)
10	12,000	−80	7.4	Serum and urine samples were collected from chronic obstructive pulmonary disease patients (n = 32) and healthy controls (n = 21), respectively. Samples were analyzed by high resolution 1H NMR (600 MHz).	(Wang et al. 2013)
NM	NM	−25	5.0	1H NMR-based metabonomics was used for the detection and diagnosis of inborn errors of metabolism from urine samples. 1D 1H NMR spectra from 47 normal, 9 phenylketonuric newborns and 1 maple syrup urine disease child were obtained and investigated.	(Constantinou et al. 2005)
NM	NM	−20	NM	The aim of this study was to assess the feasibility and comparability of metabonomic data in clinical studies conducted in different countries without dietary restriction. A group of healthy British subjects (n = 120), and healthy subjects from two European countries (Britain and Sweden, n = 30) were compared. The subjects were asked to provide single, early morning urine samples collected on a single occasion	(Lenz et al. 2004)

NM indicates not mentioned parameters

To identify the key factors that contribute to the variations in human urine metabolic profiles, we have divided this paper into two sections. In the first part of this review, we highlight and compare a number of previously published studies (Table 1) pertaining to NMR-based urine metabolomics. In the second part of this review, we propose a number of recommendations that have the potential to help minimize confounding effects, including subject selection, sample collection and sample preparation, with regard to NMR-based urine metabolomics. From this overview, we hope to help other metabolomics researchers appreciate the importance of developing global protocols to standardize the collection, preparation, storage and analysis of urine samples for the purpose of identifying disease fingerprints and biomarkers.

## NMR spectroscopy

NMR spectroscopy has played a key role in our understanding of metabolism and metabolic processes for more than three decades (Eggleston et al. 1975; Gavaghan et al. 2000). Its exceptional capacity to resolve thousands of peaks in complex metabolite mixtures such as blood and urine is probably what led NMR to be the technology of choice to initially develop the field of metabolomics (Emwas et al. 2013a; Rasmussen et al. 2012; Bouatra et al. 2013; Psychogios et al. 2011). As an analytical technique, NMR is a powerful approach for both identification and quantification of analytes with a number of important advantages, such as being highly reproducible, high-throughput, non-destructive, and non-biased, not requiring prior chromatographic separation, and, most importantly requiring minimal sample preparation (Bouatra et al. 2013; Monteiro et al. 2013; Zhang et al. 2013; Abuhijleh et al. 2009; Blindauer et al. 1997; Di Gangi et al. 2014). In addition, unlike GC–MS (Bouatra et al. 2013; Huang et al. 2013a; van der Kloet et al. 2012; Abbiss et al. 2012; Kotlowska et al. 2011; Kuhara et al. 2011) and certain liquid chromatography–mass spectrometry (LC–MS) methods (Bouatra et al. 2013; Huang et al. 2013; Lu et al. 2013; Norskov et al. 2013; Boudonck et al. 2009; Gowda et al. 2009; Tsutsui et al. 2010), no chemical derivatization or ionization are necessary. NMR is particularly amenable to detecting polar and uncharged compounds, such as sugars, amines or relatively small, volatile compounds (such as formic acid, formaldehyde, acetone, etc., which are often undetectable by LC–MS methods. Finally, multidimensional and heteronuclear NMR techniques can be employed for the unambiguous identification and characterization of unknown compounds (Abuhijleh et al. 2009; Vestergren et al. 2012; Bubb 2003; Mattar et al. 2004). The inherent low sensitivity of NMR, however, restricts the detection limit to about 1 µM and often necessitates the use of relatively large sample volumes (~200–500 µL). New micro-coil probes can reduce the sample volume to as low as a few µL (Serkova et al. 2009; Krojanski et al. 2008; Grimes and O’Connell 2011; Sukumaran et al. 2009). Nevertheless, for urine, the sample volume is generally not a problem. Overall, the numerous benefits of using NMR far outweigh its sensitivity limitations. As a result, NMR is now being used in clinical applications as well as medical research including disease diagnosis, drug assessment and personalized medicine (Emwas et al. 2013a; Nageeb et al. 2013). NMR is also being widely used in metabolite-based biomarker discovery of several human diseases (Farshidfar et al. 2012; Nahon et al. 2012; Mehrpour et al. 2013; Diaz et al. 2013; Atzori et al. 2010; Culeddu et al. 2012; Ala-Korpela 2007; O’Connell 2012; Nevedomskaya et al. 2012; Sachse et al. 2012).

The technology behind NMR spectroscopy is quite mature and most technological or equipment advances over the past decade have been relatively incremental. The developments in instrumentation, such as higher magnetic field magnets (Gruetter et al. 1998), cryogenically cooled probes (Keun et al. 2002), and microprobes (Grimes and O’Connell 2011; Sukumaran et al. 2009), are some major advancements that have enhanced the resolution and sensitivity of the method. Another recent technological development of note is called dynamic nuclear polarization (DNP). DNP provides significant increases in the sensitivity of NMR spectra (Ardenkjær-Larsen et al. 2003) and has been used to measure very low-abundance metabolites in several biological samples (Day et al. 2007; Emwas et al. 2008; Chekmenev et al. 2009; Ludwig et al. 2010). In particular, Frydman et al. has developed a new method to facilitate the use of DNP in NMR-based metabolomics research (Harris et al. 2011). However, DNP metabolomics, although intriguing as a technical improvement, is still far from the reliability and accessibility needed for routine analysis.

Although numerous types of NMR experiments are readily available, simple one-dimensional (1D) proton (^1^H) NMR spectroscopy has become the standard data collection technique for most NMR-based metabolomics studies. As a data collection method, it is fast, simple, highly reproducible and readily yields spectra that contain hundreds or even thousands of peaks. However, the main limitation of 1D ^1^H NMR spectroscopy is the fact that it generates spectra with many overlapping signals. These are due to the limited spectral dispersion of ^1^H chemical shifts, which can lead to uncertainties in the assignment and quantification of many metabolites. Other methods, such as one-dimensional carbon (^13^C) NMR spectroscopy, exhibit much greater spectral dispersion compared with ^1^H NMR spectroscopy. As a result ^13^C NMR has been proposed as an alternative method that can help overcome the spectral overlap problem of ^1^H NMR (Mavel et al. 2013; Faiz et al. 2011; Rai and Sinha 2012). However, the low natural abundance of ^13^C nuclei (1.1 %) and the low gyromagnetic ratio contribute to the relatively low sensitivity of this nucleus and hinder the widespread use of ^13^C in NMR-based metabolomics applications.

## The human urine metabolome

A comprehensive characterization of the human urine metabolome has recently been published (Bouatra et al. 2013). Extensive literature reviews combined with extensive experimental studies conducted across a variety of metabolomic platforms (e.g., NMR, HPLC, GC–MS etc.) found that more than 4,000 metabolites have been detected in urine and that slightly more than 200 metabolites can be routinely measured by NMR. Interestingly, of all the platforms and technologies assessed in this study, NMR was found to be the best—both in terms of coverage and metabolite quantification. As noted in this study, one of the main challenges of studying the human urine metabolome is its inherently high variability. The metabolic composition of urine is affected by many factors, such as diet, exercise, health status, gender, age, diurnal cycles, gut microflora as well as an individual’s genetics. For example, it has been reported that the metabolic composition of human urine is influenced by geographic location and dietary practices tied to specific cultures (Lenz et al. 2004). In one study of note, NMR-based metabolomics was employed to analyze urine samples obtained from residents of Britain and Sweden (Lenz et al. 2004). Clearly detectable differences were found in the urine samples of the two sub-groups, which were clearly attributable to dietary differences between the two countries. This result demonstrates the need to account for dietary data in urine metabolomic studies and to the need for dietary data to be used in the interpretation of any urine metabolomic study conducted for diagnostic biomarker discovery. However, even if the same individual is subjected to a strong, day by day variability due to diet and lifestyle, multiple urine collection allow one to average out this variability and to define the individual invariant part of the metabolome (Assfalg et al. 2008; Bernini et al. 2009).

## Factors leading to changes in the type and levels of metabolites in human urine

### Diet effects

Because human urine is essentially a filtrate derived from food, drink, endogenous metabolism, drugs, and other products, different nutritional habits and host/guest interactions with the gut microbiome lead to different metabolic signatures in urine. Application of metabolomics to the discovery of new dietary biomarkers has been extremely fruitful in recent years, highlighting the important influence of dietary factors on urinary profiles (Heinzmann et al. 2010; Lloyd et al. 2011). Over the past 15 years, several NMR-based metabolomic studies have been conducted to systematically explore the effects of different diets and dietary components on the chemical composition of urine (Walsh et al. 2006; Walsh et al. 2007; Stella et al. 2006; Llorach-Asuncion et al. 2010; Llorach et al. 2009; Holmes et al. 2008a; Fave et al. 2011; Winnike et al. 2009). In one particularly important study, ^1^H NMR spectroscopy was employed to study urine samples collected from ten healthy volunteers who were admitted to a clinical research center for 2 weeks of dietary standardization (Winnike et al. 2009). The results suggested that 24 h of dietary standardization could be enough to provide the normalization necessary in a clinical research setting (Winnike et al. 2009). In another study, the results of analyzing the urine of 30 healthy individuals revealed that the metabolic profile in urine is more sensitive than other biofluids (plasma and saliva) to acute dietary intake (Walsh et al. 2006). These results show that dietary variation has the potential to be a significant confounding factor in metabolic profiles of urine and may interfere with disease biomarker studies. More recently an NMR-based metabolomics study demonstrated that dietary patterns were clearly reflected in urinary metabolites, where *O*-acetylcarnitine was positively associated with red-meat consumption while phenylacetylglutamine associated with vegetable intake (O’Sullivan et al. 2011).

In a comprehensive study published in 2006, Nicholson et al. combined NMR-based metabolomics with multivariate statistical analysis to study the effects of three diets (mostly meat, minimal meat and vegetarian) on human metabolic phenotypes (metabotypes; see later) (Stella et al. 2006). This study was conducted with a long dietary standardization period during which 12 healthy participants consumed each diet continuously for 15 days followed by 7 days washout periods. Proton NMR spectra of the urine samples collected from each participant on days 13, 14 and 15 were recorded under the same experimental conditions. The results revealed the different urine metabolic signatures of different diets. Higher levels of some metabolites, such as carnitine, acetylcarnitine, and trimethylamine-*N*-oxide (TMAO) were found to be associated with a mostly meat diet (Stella et al. 2006). Furthermore, the urinary concentration of creatine (a metabolite that is found mainly in muscle tissue) was much higher in samples taken during the mostly meat diet than in samples from the minimal meat and vegetarian diets. On the other hand, *p*-hydroxyphenylacetate (a microbial/mammalian co-metabolite) was elevated in the samples taken from those on a vegetarian diet compared with samples taken from those on meat diets. This indicates that urine is sensitive to alterations of bacterial composition or metabolism in response to diet (Stella et al. 2006). The most significant results of this study were found in both the inter-individual and the intra-individual differences in the metabolic signatures of urine, with some participants showing a much greater metabolic response to diet standardization than did other participants. This confirms the effect of an individual’s metabotype on his/her urinary metabolic fingerprint that has to be considered in urine biomarker investigations.

### Effects of sample collection time

In a comprehensive study published in 2007, the effect of the time of day on urine collection was evaluated (Slupsky et al. 2007). ^1^H NMR spectra of urine samples collected in the morning were compared with proton NMR spectra of samples collected from the same subjects in the afternoon. The concentrations of some metabolites, such as creatinine, mannitol, dimethylamine, 1-methylnicotinamide, xylose, and acetone, differed between the samples collected in the morning versus the samples collected in the afternoon (Slupsky et al. 2007). In another study, it was found that the concentration levels of 4-aminohippurate, aspartate, creatine, formate, glutamate, phenylalanine, salicylurate, tryptophan, and transaconitate were found to be different between morning and afternoon urine collection (Saude et al. 2007). The effects of collection time on metabolite concentration levels in human urine have been reported by other studies, suggesting that the sample collection time should be standardized in any urine metabolomics study (Maher et al. 2009; Saude et al. 2007). In general, it is reasonable to collect urine samples in the usual cups or vacutainers used for the common clinical urinalysis. To avoid potential chemical contaminant variations coming from the containers or filters, ideally one should try to use the same kind/brand of containers and filters for the duration of a given project.

### Age and gender effects

Proton NMR spectroscopy was employed to study the effect of gender and age on the metabolic composition of urine (Kochhar et al. 2006; Faughnan et al. 2004; Gavaghan et al. 2000). In one study, the age of 40 was chosen as a threshold between young and old. The results demonstrated that proton NMR spectra can distinguish young and old subjects, suggesting that age plays a determining role in urine metabolite levels. Several metabolites, such as carnitine, 3-hydroxyisovalerate, creatinine, alanine, and trigonelline, could be used to distinguish the samples from young and old subjects (Slupsky et al. 2007). In another study, Kochhar et al. used NMR spectroscopy to analyze urine samples from 150 healthy participants to investigate the effects of gender, age and body mass index (BMI) on the metabolic signatures found in human urine. The results showed that the metabolic signatures in urine could be distinguished based on gender, age and BMI. Taurine, creatinine, and citrate could distinguish the gender of the subjects with taurine and creatine/creatinine concentrations being more elevated in urine from males, whereas citrate concentrations were elevated in the urine from females (Kochhar et al. 2006). Glycine, citrate, creatine/creatinine, dimethylamine, and other unidentified metabolites could be used to distinguish the age of the subjects. Gender metabolomic signatures in urine have also been demonstrated in other metabolomics studies (Duarte and Gil 2012; Faughnan et al. 2004; Psihogios et al. 2008).

### Human metabolic phenotypes (metabotypes)

Determining an individual’s unique personal phenotype could be particularly useful in clinical and therapeutic applications such as personalized medicine and for following disease progression and enabling early diagnosis and prevention. Individual phenotypes may be different not only because individuals are genetically different, but also because of different environmental factors. Thus, the phenotype could be more relevant than the genotype when dealing with disease pathologies and individual responses to drug intervention or other external environmental stimuli. From an NMR-based metabolomics study of animal urine samples, Gavaghan et al. proposed the existence of unique metabolic phenotypes termed metabotypes (Gavaghan et al. 2000). A metabotype was defined as “a probabalistic multiparametric description of an organism in a given physiological state based on analysis of its cell types, biofuids or tissues” (Gavaghan et al. 2000).

By means of repeated sampling at different days in the same individual (multiple samples approach), Assfalg et al. provided evidence that individual phenotypes do exist. They investigated 873 human urine samples collected from 22 healthy subjects over 3 months (Assfalg et al. 2008). Samples collected from a subset of the same individuals 2 and 3 years after the initial study provided additional proof of the existence of individual metabotypes and showed that the metabolic fingerprint is stable over a time period of at least 2 or 3 years (Bernini et al. 2009; Blindauer et al. 1997). Furthermore, anecdotal evidence was reported indicating that identical twins can share very similar metabolic phenotypes (Bernini et al. 2009). Furthermore, the results demonstrated that an individual’s metabolic phenotype is affected by both the gut microbiome and host metabolic metabotypes (Bernini et al. 2009).

The multi-sample approach for characterizing an individual’s metabolic fingerprint opens the door to a new approach for biomedical research. In particular, whereas the metabolic snapshot (single sample) approach is generally used to study correlations between pathologies and the metabolome by comparing the differences between the metabolites found in a group of healthy individuals and a group of patients, the multiple-sample approach can be used to more accurately assess inter-person variability. It can also be used to take into account variations linked to disease development or progression, changes in lifestyle or stress response. In this way, an individual can act as a control for him/herself, increasing the power of the analysis. Multi-sample collection from the same individual over a long period of time will allow following disease onset/progression and the evaluation of the individual response to therapeutic intervention.

### Gut microflora effects

The human body is known to be a host for numerous and complex consortia of microorganisms, many of which are found in the gut (Nicholson et al. 2012; Blumberg and Powrie 2012; Holmes et al. 2012). It has been reported that the development of an individual’s microbiome starts at birth when the microbial landscape (seed ecology) is passed from mother to child. After birth, it starts to take shape, subject to several factors, such as diet, lifestyle, use of antibiotics and pathological states (Ravel et al. 2011; Torrazza and Neu 2011; Nicholson and Wilson 2003). For example, intestinal bacteria are associated with the pathogenesis of several diseases, including irritable bowel syndrome (Martin et al. 2006), cardiovascular disease (Pereira and Gibson 2002), insulin resistance (Dumas et al. 2006), Crohn’s disease, gastrointestinal cancer (Dunne 2001), and celiac disease (Tjellstroem et al. 2007). Gut microflora also play a crucial role in regulating key physiological activities, such as processing and absorbing nutrients and the metabolism of many xenobiotic compounds (Lee et al. 2012). Several studies have shown that high levels of hippuric acid, and many other aromatic compounds observed in the urine of animals, originate from gut microbial metabolism of dietary polyphenols (Nicholls et al. 2003; Williams et al. 2002). It was pointed out that the gut is one of the important factors in determining the host’s metabolic phenotype (McKee et al. 2006). Since the outcomes of treatment interventions are influenced by an individual’s metabolic phenotype for which the gut status is a determining factor (Dumas et al. 2006), it has been proposed that gut microorganisms should be considered as part of personalized treatment solutions (Holmes et al. 2008b; Clayton et al. 2006; Nicholson et al. 2005). Recently, NMR-based metabolomic approaches were used to study the endogenous metabolic changes in the urine of pseudo germ-free rats. Changes in the levels of 25 metabolites were associated with the activities of gut microflora, highlighting the importance of gut microflora in the metabolic composition of urine in disease-related studies. All of the affected metabolites could be potential biomarkers (Lee et al. 2012). Recent studies (Bertini et al. 2009, 2011a) showed that, by applying NMR-based metabolomics to the study of celiac disease, several urinary metabolites originating from gut microflora were found to be significantly different between healthy controls and celiac patients.

### Physical activity

Human metabolism is directly associated with physical activity. Such activity can affect the metabolic signature of urine over both short and long time periods. ^1^H NMR-based metabolomics approaches have been used to study metabolomic modifications in urine after different kinds and levels of physical activities (Enea et al. 2010). The results showed that it was possible to distinguish between urine samples collected before and after exercise, with levels of lactate, pyruvate, alanine, β-hydroxybutyrate, and hypoxanthine increased after exercise (Enea et al. 2010). ^1^H NMR-based metabolomics combined with multivariate statistical analysis could also differentiate the urine metabolome of participants involved in two exercise sessions which differed in the duration of the rest interval between repeated exercise efforts, namely three sets of two 80 min maximal runs separated by either 10 s or 1 min rest times (Pechlivanis et al. 2010). Samples collected pre and post exercise could be differentiated based on the metabolites lactate, pyruvate, hypoxanthine, compounds of the Krebs cycle, amino acids, and products of branched-chain amino acid (BCAA) catabolism whilst samples from the different rest intervals were separated via lactate, pyruvate, alanine, compounds of the Krebs cycle, 2-oxoacids of BCAA, and 2-hydroxybutyrate with higher amounts of these metabolites in samples from the shorter rest time. The authors attribute these increased metabolite levels to the greater metabolic disturbances arising from very limited recovery time (10 s) between each run, and highlight the potential role of metabolomics in enhancing knowledge of exercise physiology.

### Interactions between factors that affect metabolite levels

The factors that affect metabolite concentration levels in human urine can be divided into two categories. The first category represents the factors that cannot be controlled such as age, gender, metabotype and gut microflora while the second category represents the factors that can be controlled such as diet, sample collection methods, sample storage and sample preparation and therefore require standardization. However, these factors are not completely independent. Indeed they can significantly influence each other, which further complicates disease diagnostic studies. For example, the level of hippurate, which is one of the most abundant metabolites in human urine, can be influenced by a wide range of physical, emotional and dietary effects. Substantial variations in hippurate levels have been associated with different psychiatric disorders disorders with decreased levels in schizophrenia and depression and elevated hippurate excretion levels during episodes of anxiety (Quastel and Wales 1938; Fabisch and Fellner 1957; Johannsen et al. 1962; Persky et al. 1950). Increased levels of hippurate were also found in the urine of type-I diabetic patients compared with samples from healthy controls (Zuppi et al. 2002). In another study, lower levels of hippurate were detected in urine samples from 15 morbidly obese subjects compared with samples collected from 10 healthy age-matched controls (Calvani et al. 2010). Perturbations in urinary hippurate levels were also reported in urine samples of children with autism (Yap et al. 2010). The perturbation of hippurate concentrations in human urine can be confusing in novel disease biomarker identification or validation as several diseases may lead to similar changes. Moreover other unrelated cofactors can also lead to different changes in the hippurate level. For example, elevated hippurate excretion has long been associated with several dietary components including tea, coffee, fruits and vegetables (Clifford et al. 2000; Cathcartrake et al. 1975). NMR-based metabolomics studies have also revealed significant changes in hippurate concentration levels in the urine of healthy subjects and these changes correlated with the subject’s age (Psihogios et al. 2008). Variation in the excretion levels of hippurate has also been found to be gender-correlated where higher hippurate concentrations were reported in the urine of females compared with age-matched male subjects (Psihogios et al. 2008; Siqueira and Paiva 2002). The effects of gut microbiota on hippurate concentrations in human and animal urine samples have been frequently reported (Nicholls et al. 2003; Nicholson et al. 2005; Cotran et al. 1960). Moreover, these cofactors may influence each other when different diets affect the gut microbiota excretions of hippurate (Lees et al. 2013), leading to further complications in disease diagnostic studies. Given these many influences, it is probably safe to say that hippurate cannot reasonably be used as a novel biomarker for any condition.

## Sample preparation and experimental conditions

There are many reasons for modified metabolic concentrations in human urine, including diet, drug administration, health conditions, physical activity and environmental stressors. The collection of multiple samples from the same donor over a relatively extended time period helps identify the invariant part of the urine metabolome that characterizes an individual’s metabotype (Gavaghan et al. 2000; Bernini et al. 2009; Assfalg et al. 2008). On the other hand, the pre-analytical treatment of urine samples may alter the original metabolic profiles (Bernini et al. 2011b). The major cause of pre-analytical variation in urine samples arises from the presence of human or bacterial cells that may break upon sample freezing (due to water crystal formation) or arise from harsh centrifugation conditions employed to remove particulate matter from urine samples used in NMR. If cells are eliminated by filtration (or mild centrifugation) before NMR sample preparation or long-term storage, the unwanted effects can be easily attenuated. Adding NaN3 to urine at the moment of collection, or just before freezing the samples, is a recommended step in many proteomics analysis protocols to avoid bacterial growth (Thongboonkerd et al. 2006; Thomas et al. 2010) In NMR metabolomic studies it is more common to store samples without any preservative agent and to add sodium azide at the time of sample preparation for NMR analysis (Saude and Sykes 2007). However, there are examples of metabolomic studies where sodium azide was added immediately after sample collection (Grison et al. 2013; Ganti and Weiss 2011). It is well known that the presence of bacteria can alter the metabolic profile of urine (Saude and Sykes 2007), consequently it is recommended that urine samples be collected midstream (thereby avoiding the early urine flow that contains urethral contaminants) (Lewis et al. 2013) which is also the standard way to collect urine samples for clinical urinanalysis (Delanghe and Speeckaert 2014).

### Subject (sample) selection

While most metabolomic studies pay attention to factors such as age, gender and diet, little information if any is available about the criteria for the choice of healthy control subjects. For comparative purposes, it is important that the definition of normal or healthy subjects be uniform. While there is a clear difference between someone being healthy and someone simply lacking disease symptoms, in most studies, a healthy subject is considered to be an individual with no underlying chronic disease. Beyond ensuring comparable ages and genders, healthy subjects should also share the same ethnicity and BMI as well as other environmental factors, such as geographical location, diet and whenever possible, lifestyle (e.g., level of exercise).

Apparently healthy (or disease-free) individuals frequently take dietary supplements, herbal medicines or over-the-counter drugs. These can clearly affect the metabolic composition of the urine. Therefore, a well-designed study should track consumption of these apparently “innocuous” compounds. In some cases, evidence of these compounds can be detected in the urine; however, without reference spectra for the compounds, their identity is often unknown. Finally, is worth mentioning that an individual can effectively act as his/her own control. This approach should encourage the development of metabolomics baseline screening as a routine procedure for large populations. When an individual is compared with his/herself, especially before the onset of the disease or before an intervention study, the variability is largely reduced, and the validity of the statistical analysis is improved (Westerhuis et al. 2010; Jansen et al. 2005). Careful attention should also be paid when collecting samples from patients who suffer from other diseases or with severe health conditions. As a general rule, no sample should be considered from “diseased” subjects who suffer from other obvious diseases apart from the target disease of interest.

### Sample storage

Metabolomics researchers use a wide variety of methods and protocols to store urine samples. Many use freezing for long-term storage but the storage conditions may vary between different laboratories and even within the same lab or the same study. The effect of storage conditions on the metabolic composition of urine has been extensively studied using proton NMR-based metabolomics (Lauridsen et al. 2007). The results from this study showed that metabolite levels in human urine samples that were stored at or below −25 °C did not change during 26 weeks of storage time. On the other hand, formation of acetate was observed in some urine samples stored at 4 °C without the addition of any preservation agent. These changes were apparently due to microbial contamination because different urine samples have different microbial compositions (Lauridsen et al. 2007). The use of a 0.22 µm filter was recommended as a further step to separate urine from bacterial contaminants (Lauridsen et al. 2007; Gika et al. 2007; Saude and Sykes 2007; Barton et al. 2008). It is generally recommended that investigators use filtration and/or gentle centrifugation before freezing their urine samples to avoid the breakage of human or bacterial cells that may be present in the sample (Bernini et al. 2011b). Release of enzymes from cells induces changes in the metabolite composition and pH over time. Adding a preservative agent such as sodium azide (0.01–0.1 %) is also commonly used prior to urine storage because it can have an inhibitory effect on residual bacterial or enzymatic activity. For comparative studies, it is crucial to use identical types of collection tubes, filters and storage procedures according to an optimized standard operating procedure (SOP). Indeed, several reports have shown that microbial contamination and sample storage at 4 °C would lead to significant changes in the metabolic profile of urine samples and thus standardization of collection and storage of urine samples is strongly advised (Rasmussen et al. 2011; Ryan et al. 2011; Saude and Sykes 2007). It is also known that some urine analytes are sensitive to light. In the clinical literature, amber-colored urine containers are occasionally recommend to protect specimens from light, especially for certain kinds of urine tests (Delanghe and Speeckaert 2014). To the best of our knowledge, no systematic evaluation of the effect of the light on the urinary metabolome has been performed. To avoid repeated freezing/thawing cycles and repeated long-term exposure to light, urine samples should be split and stored as multiple, small aliquots (Bernini et al. 2011).

### Salt and pH optimization

Depending on the person’s acid–base status, the pH value of normal human urine ranges from 5 to 8 (Martin Hernandez et al. 2001; Rylander et al. 2006; Welch et al. 2008). Compounds with chemical groups (such as imidazoles) that have pKa values near the physiological range are particularly sensitive to pH changes. Consequently, pH changes can lead to significant chemical shift changes in the NMR signals of these molecules (Xiao et al. 2009). Likewise, some polyvalent carboxylates (such as citrate) are particularly good at chelating metal cations. Consequently, these compounds exhibit significant chemical shift changes as the salt concentrations change (Bernini et al. 2011b; Foxall et al. 1992). Thus, it is crucial to adjust the pH of all urine samples prior to any NMR measurements. In many studies, a phosphate buffer is frequently used to maintain the desired pH value (Bernini et al. 2011b; Assfalg et al. 2008; Bernini et al. 2009). In one recent study, it was found that even with a concentration of 150 mM phosphate at pH 7.0, the pH values of urine vary approximately between 6.8 and 7.2 (Rist et al. 2013). Different approaches can be implemented to overcome the residual pH variation problem including the use of higher buffer concentrations (Lauridsen et al. 2007) and adjusting the pH by adding a small volume of acid, such as HCl, or a small volume of a concentrated base, such as NaOH (Bertram et al. 2007b). However, forcing the urine’s pH to a constant value can hide physiological or pathological information encoded in the pH changes. To eliminate the variations on the chemical shift resulting from inter-sample differences in divalent cation concentrations such as (Ca^+2^ and Mg^+2^), pre-precipitating with KF followed with adding deuterated EDTA was proposed (Jiang et al. 2012). It has been also recommended to use K_2_HPO_4_ instead of Na_2_HPO_4_ where high concentrations of buffer are needed to overcome the low solubility of Na_2_HPO_4_. In this case one can use buffer-urine ratio of 1:10 instead of traditionally 1:2, with better signal to noise ratio (Xiao et al. 2009).

If the ultimate goal is to move research from the benchtop to clinical practice, metabolomics researchers need to use well-standardized experimental conditions including standardized or optimized pH values. To avoid any sample preparation and/or instrumental variation that may lead to chemical shift differences, it is also highly recommended that a standard reference for chemical shift calibration be added. For proton and carbon NMR measurements of urine samples, both 4,4-dimethyl-4-silapentane-1-sulfonic acid (DSS) or its sodium salt and 3-trimethylsilylpropionic acid (TSP) can be used. DSS is known to be much less pH sensitive, but it is somewhat more hydrophobic than TSP. This hydrophobicity can lead to DSS binding (to proteins or lipids), which broadens the DSS reference line.

### Experimental temperature

Temperature is another important factor affecting chemical shifts in metabolites, particularly in metabolites containing amide groups. Amide groups can form hydrogen bonds, and when the temperature is changed, the NMR chemical shifts of amide groups and the adjacent protons can vary, causing problems for later data analysis. In addition, temperature also impacts the T_1_ and T_2_ relaxation of metabolites. T_2_ relaxation is associated with the line-width of NMR signals, whereas T_1_ relaxation is related to NMR signal intensities. Hence, temperature variation can lead to problems in the quantification of metabolites. Therefore, in terms of data quality, it is vital to keep the temperature constant in all NMR acquisitions. It is usually convenient to keep the temperature at 25 °C (298 K). To ensure temperature equilibration samples should be left in the NMR probe for 10 min prior to spectral acquisition; a longer time is needed if an automated sample exchanger with cooling rack is used.

## The metabolomics society and standardization initiatives

Several parallel metabolomics standardization efforts started as early as 2004, and by 2007 these efforts were gathered under the umbrella of the Metabolomics Society and rebranded as the Metabolomics Standards Initiative (MSI) (Salek et al. 2013c; Fiehn et al. 2007; Sumner et al. 2007). One major outcome was standardization on reporting metabolomics experiments with a set of minimum information guidelines, including a reporting requirement for NMR-based experiment data acquisition. In 2012, an FP7-funded EU Initiative called coordination of standards in metabolomics (COSMOS) was set up to bring together leading researchers and bioinformaticians of the European metabolomics community, members of the Metabolomics Society, along with other stakeholders worldwide to develop data infrastructure guidelines and workflows for a broad range of metabolomics applications (www.cosmos-fp7.eu). COSMOS also realizes that the potential of metabolomics cannot be exploited without major standardization of formats and terminologies. To work on commonly agreed-upon metabolomics data standards, the COSMOS initiative has gathered metabolomics and bioinformatics experts to establish a common data exchange format (syntax) and data semantics that maximize interoperability with other omics standards. This is achieved by using the general-purpose Investigation/Study/Assay tabular format (ISA-Tab) (Rocca-Serra et al. 2010) for experimental meta data information and adapting the xml-based formats for the instrument derived “raw” data types by the Proteomics Standards Initiative (PSI) (Orchard et al. 2003; Hermjakob et al. 2003). Data completeness is verified by using and extending the MSI Core Information for Metabolomics Reporting (CIMR http://biosharing.org/bsg-000175).

## Recommendations

Based on an extensive review of the literature (summarized, in part, here) as well as discussions and agreements reached among the authors we wish to propose the following “best practice” recommendations regarding the collection, storage and NMR analysis of urine samples for the purposes of NMR-based metabolomics.

- *Ethical guidelines* Before collecting any urine sample, ethical approval from the local research committee should be obtained. All donors must read and sign an informed consent, according to the ethical guidelines and privacy regulations associated with a given institution. As the ultimate goal is to generate reproducible data that can be exchanged between different groups it would reasonable to have a common ethical guideline proposed by Metabolomics Society, stating that donors are aware and they have agreed on sharing their sample data between different groups for research purposes.
- *Standard operating protocols* (*SOPs*) The NMR metabolic fingerprint of sera/plasma and urine samples was monitored to establish the optimal standard operating procedures (SOPs) for pre-analytical handling of these biofluids for metabolomic studies and biobanks (Bertini et al. 2011b). The authors proposed the following procedures for the optimal processing and management of urine samples: (1) removal of cells and particulate matter through the combined use of a mild pre-centrifugation step at 1,000–3,000 rpm (5 min at 4 °C) using a 0.22 micron filter; (2) long-term storage of samples in liquid nitrogen (or liquid nitrogen vapor) to avoid the lysis of residual cells; (3) fast processing (within 2 h of collection); and (4) storage at 4 °C between collection and processing. In the opinion of the authors, the addition of additives (such as enzyme inhibitors) should be avoided because the required concentrations introduce unwanted signals in the NMR spectra that would mask the resonances of metabolites of interest and may also induce changes in pH, ionic strength, etc. that affect the original NMR profiles.
- *Consumables* use one brand of consumables for all samples (e.g., containers for sample collection and storage, sample preparation tubes, filtration devices and NMR tubes). Note that collection and storage tubes as well as filtration units may contain impurities such as polyethylene glycol, so they should be screened to establish their purity prior to use.
- *Sample or patient selection* detailed criteria for sample/patient selection should be included in the Methods section or the experimental protocol. For example, samples collected from patients with other diseases or confounding conditions (such as kidney failure, diabetes and any metabolic disorders) apart from the target disease should be excluded from diagnostic studies. If possible, good quality dietary, dietary supplement and drug intake information should also be collected for each individual. Dietary information may be obtained through food frequency questionnaires, while drug intake and dietary supplement information may be obtained through direct patient queries. The minimum information required from each participant for diagnostic investigations (age, gender, ethnicity, BMI, smoking status, etc.) is proposed in Table 2.

**Table 2:** Subject information that is proposed to be included in diagnostic studies

Phenotypic descriptions	Genotype information	Individual information	Environmental factors
Height	Gender	Pregnancy, menstruation	Living area
Weight	Ethnicity	Restricted diet (for example vegetarian, vegan etc.)	Health status, Mental status
Body mass index	Progeny	Smoker	Nutrition (food intake)
Waist–hip ratio*	Single nucleotide polymorphisms (SNPs)**	Alcoholic	Drug administered
Other	Other	Lifestyle (work)	Physical exercise
		Other	Fasting and others

* Waist–hip ratio is significant factor in studies that involve obesity and obesity related disease such as diabetes and cardiovascular (de Koning et al. 2007; Czernichow et al. 2011; Chan et al. 2003; Shah et al. 2009)

** (SNP) Single-nucleotide polymorphism
- *Sample collection* urine should be collected in the morning (preprandial). To avoid contamination from urethral bacteria, only mid-stream urine should be collected. At least 15 mL of urine should be collected in a sterile polyethylene tube. Sample tubes must be leak-proof and tightly sealed. The samples should be processed and aliquoted within 2 h from the collection, but preferably faster. Samples must be kept refrigerated at 4 °C prior to processing and must not be frozen prior to processing (Bernini et al. 2011b).
- *Centrifugation* urine samples should be centrifuged at 1,000–3,000 rpm (5 min at 4 °C) and then filtered using a 0.22 µm filter before aliquoting and storage (Bernini et al. 2011b) using appropriately labeled cryovials
- *Additives* ideally, urine samples should be free of externally added chemicals or enzymes to reduce interference with metabolite signals. However, addition of micromolar quantities of inorganic bacteriostatic agents such as sodium azide (to limit bacterial growth) is appropriate and justified. The addition of small (~1 mM) amounts of EDTA (Jiang et al. 2012) has also been shown to reduce paramagnetically induced chemical shift variability among certain compounds, thereby giving greater spectral reproducibility.
- *Sample freezing* dry ice should not be used to freeze urine samples (Rist et al. 2013). For short-term storage (<2 weeks), samples can be stored at −20 °C. For long-term storage, urine samples should be stored at −80 °C using an appropriate freezer. If possible, for very long-term storage, it is better to keep the samples in liquid nitrogen vapor (Bernini et al. 2011b).
- *Sample transfer* if samples must be transferred or re-aliquoted, it is important to maintain the cold chain during transport, storage and delivery using appropriate cryogenic storage dewars.
- *Chemical shift referencing and locking* small amounts (<1 mM) of DSS (preferred) or TSP (second choice) should be added to urine samples prior to NMR spectral acquisition. 5–10 % D_2_O (as part of the pH buffer) should also be added to all urine samples to permit signal locking.
- *pH* urine samples should be appropriately buffered with a non-organic buffer (i.e., 100–150 mM phosphate) and brought to a pH between 6.8 and 7.4. Details of the buffering process and pH adjustment protocol should be provided. If samples must be analyzed at very low pH values (to detect certain compounds) or without modification of the pH, this information must be provided and appropriately justified.
- *Sample numbers* unlike animal models or cell line experiments, where experimental and environmental conditions can be well controlled, human studies do not have the same controls. Consequently, larger numbers of samples are necessary to mitigate inter- and intra-sample variations. The number of samples used in different studies can vary significantly, depending on the strength of differentiating signals. Ideally, the number of the samples used should be justified during the experimental planning phase, using appropriate power calculations. However, in exploratory or pilot studies where statistical information for power calculations is not available, a good rule of thumb is to use ~30 samples and ~30 controls.
- *Sample randomization* If larger numbers of samples are being studied (>10), spectral acquisition should be performed after sample randomization, in order to avoid biasing results due to instrument conditions or operator differences.
- *Magnetic field* although NMR-based metabolomics studies on urine would benefit from the use of the highest accessible fields, to date most NMR-based metabolomic studies have been conducted using 600 MHz NMR spectrometers, as these instruments offer a good compromise between sensitivity, resolution and cost.
- *Pulse sequences* most applications of NMR for metabolomics research rely on 1D NMR experiments. The technique used for water suppression is critically important for the comparability between NMR data. Results obtained from multivariate analysis of NMR spectra taken under different solvent suppression conditions can be erroneously interpreted as biological differences (Potts et al. 2001) if water suppression artifacts are not carefully excluded. The most used 1D NMR pulse sequence in urine metabolomics is 1D-noesypresat (Kumar et al. 1980). Longer acquisition times (4 s) and shorter delays (~1 s) appear to produce the best results regarding maximal metabolite signal intensity and minimal water signal. Shimming: While automated shimming options in new NMR spectrometers have made metabolomic data collection relatively quick and easy, manual shimming might be necessary to improve the quality of spectra. Adjustment of the field and lock position/phase during the shimming process should also improve the signal quality (signal-to-noise, peak symmetry and line width). The shims can be optimized by assessing line shape and width at half height of the reference signal, where the line width of standards such as DSS or TSP should yield a signal between 0.5 and 1.0 Hz.
- *Temperature* it is known that variation of experimental temperature can affect the chemical shift of metabolites, to differing extents (Hongting Cao 2008). Thus, it is crucial to standardize and maintain the NMR experimental temperature. We propose 298 K as the standard temperature, which is close to average room temperatures such that the experimental temperature may be easily maintained during the course of the experiment when many samples are usually involved. To ensure temperature equilibration, samples should remain in the NMR probe for 10 min before spectral acquisition and a longer time is needed when an automated sample exchanger with a cooling rack is used for loading a sequence of samples.
- *Minimum experimental and instrumental reporting standards* for the sake of creating comparable and exchangeable NMR data between different groups, it is important to standardize the minimum experimental details including sample selection, collection, storage and preparation along with reporting NMR parameters as proposed by the Metabolomics Standards Initiative (Sumner et al. 2007). This enables the interrogation and comparison of NMR data as well as facilitating experimental replication (Sumner et al. 2007).
- *Reporting new bio-markers* In addition to careful consideration of all factors that may influence metabolite concentrations in human urine, researchers should pay extra attention when reporting new disease biomarkers derived from urinary measurements. In particular, we propose that new urinary biomarkers should be absolutely quantified or quantifiable, they should be appropriately normalized (using creatinine, specific gravity or other accepted methods), they should be robust towards the identification of the disease (sensitivity and/or specificity >0.7), they should have a well-defined formula, threshold or decision algorithm that is clearly stated and the biomarker(s) should be validated using different populations (differing in geographic location, age, gender and/or ethnicity), including populations with potentially confounding disease states or symptoms.

## Concluding remarks

Human diseases lead to different variations in metabolite concentrations that are directly correlated with disease progression. For example, if the concentration of certain metabolites increases as a result of disease status, these changes can provide valuable information about the progression of the disease. Furthermore, a disturbance in metabolite levels could happen before the onset of clinical symptoms, thereby making the marker(s) useful for disease prediction. In addition to disease diagnosis, prognosis and prediction, the identification of novel disease fingerprints and biomarkers is also proving to be important in increasing our understanding of disease pathology and in monitoring treatment efficacy. Despite the general success of using NMR-based metabolomics of urine in distinguishing between healthy and diseased individuals, novel biomarker discovery remains an on-going challenge. Several factors such as lifestyle, diet, ethnicity, general health condition, sample selection, sample storage, and sample preparation can alter metabolite concentrations in urine, leading to major problems that may complicate and interfere with a study. In an effort to mitigate these problems, we have conducted a broad survey of the literature and investigated (within our labs) some of the current practices for NMR-based metabolomics studies of urine. Based on this information, we have identified a number of best practices and have compiled a series of recommendations for conducting NMR-based metabolomic studies of urine. In presenting and justifying these recommendations, we hope that the metabolomics community will adopt them as part of their standard experimental and publication routine. These best-practice recommendations certainly could go a long way to help the metabolomics community translate important discoveries from the lab into the clinic.
